# Annexin-A1 and caldesmon are associated with resistance to tamoxifen in estrogen receptor positive recurrent breast cancer

**DOI:** 10.18632/oncotarget.6521

**Published:** 2015-12-09

**Authors:** Tommaso De Marchi, Anne M. Timmermans, Marcel Smid, Maxime P. Look, Christoph Stingl, Mark Opdam, Sabine C. Linn, Fred C. G. J. Sweep, Paul N. Span, Mike Kliffen, Carolien H. M. van Deurzen, Theo M. Luider, John A. Foekens, John W. Martens, Arzu Umar

**Affiliations:** ^1^ Department of Medical Oncology, Erasmus MC Cancer Institute, Erasmus University Medical Center, Rotterdam, The Netherlands; ^2^ Department of Neurology, Erasmus University Medical Center, Rotterdam, The Netherlands; ^3^ Division of Medical Oncology, Netherlands Cancer Institute – Antoni van Leeuwenhoek Hospital, Amsterdam, The Netherlands; ^4^ Department of Laboratory Medicine, Radboud University Medical Center, Nijmegen, The Netherlands; ^5^ Department of Radiation Oncology, Radboud University Medical Center, Nijmegen, The Netherlands; ^6^ Department of Pathology, Maasstad Hospital, Rotterdam, The Netherlands; ^7^ Department of Pathology, Erasmus University Medical Center, Rotterdam, The Netherlands; ^8^ Cancer Genomics Center Netherlands, Amsterdam, The Netherlands

**Keywords:** tamoxifen resistance, annexin-A1, caldesmon, clinical proteomics, metastatic breast cancer

## Abstract

Tamoxifen therapy resistance constitutes a major cause of death in patients with recurrent estrogen receptor (ER) positive breast cancer. Through high resolution mass spectrometry (MS), we previously generated a 4-protein predictive signature for tamoxifen therapy outcome in recurrent breast cancer. ANXA1 and CALD1, which were not included in the classifier, were however the most differentially expressed proteins. We first evaluated the clinical relevance of these markers in our MS cohort, followed by immunohistochemical (IHC) staining on an independent set of tumors incorporated in a tissue microarray (TMA) and regression analysis in relation to time to progression (TTP), clinical benefit and objective response. In order to assess which mechanisms ANXA1 and CALD1 might been involved in, we performed Ingenuity pathway analysis (IPA) on ANXA1 and CALD1 correlated proteins in our MS cohort. ANXA1 (Hazard ratio [HR] = 1.83; 95% confidence interval [CI]: 1.22–2.75; *P* = 0.003) and CALD1 (HR = 1.57; 95% CI: 1.04–2.36; *P* = 0.039) based patient stratification showed significant association to TTP, while IHC staining on TMA showed that both ANXA1 (HR = 1.82; 95% CI: 1.12–3.00; *P* = 0.016) and CALD1 (HR = 2.29; 95% CI: 1.40–3.75; *P* = 0.001) expression was associated with shorter TTP independently of traditional predictive factors. Pearson correlation analysis showed that the majority of proteins correlated to ANXA1 also correlated with CALD1. IPA indicated that ANXA1 and CALD1 were associated with ER-downregulation and NFκB signaling. We hereby report that ANXA1 and CALD1 proteins are independent markers for tamoxifen therapy outcome and are associated to fast tumor progression.

## INTRODUCTION

ER positive breast cancer constitutes three quarters of all breast malignancies. Treatment options of patients with such tumors include targeted anti-hormonal drugs, of which tamoxifen has been the first choice for decades, both in the adjuvant and in the recurrent setting [1]. In the adjuvant setting, tamoxifen significantly increases patient survival and decreases the risk of metastasis occurrence [2]. In recurrent ER positive breast cancer, approximately 50% of patients treated with tamoxifen manifests intrinsic drug resistance, while the other half experiences acquired resistance during therapy [3, 4]. Many studies have described mechanisms of tamoxifen resistance in breast cancer patients, such as the upregulation of ER transcriptional co-activators [5] or the expression of ER isoforms [6], the activation of several tyrosine kinase pathways such as PI3K/MAPK [7], or the dysregulation of tamoxifen metabolizing enzymes [8]. Acquired mutations in the ligand-binding domain of ESR1 protein (e.g. p.Tyr537Ser) during endocrine treatment have also been associated with constitutive agonistic activity of the receptor and the unresponsiveness to anti hormonal therapies [9–11]. Furthermore, gene expression analyses have been performed to derive biomarkers predictive of tamoxifen therapy outcome in both the adjuvant and the recurrent settings [12, 13]. So far, none of these markers have found clinical application due to non-optimal study design, lack of extensive sample validation, or difficulty in developing assays into an accurate and standardized format [14, 15].

**Figure 1:**
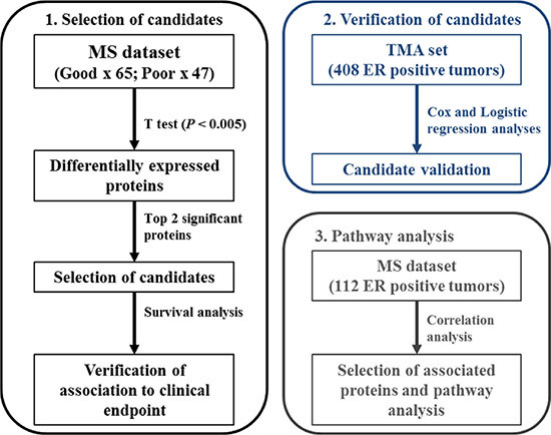
Schematic representation of analysis workflow

Proteomics-based technologies have shown to enable expansion of the depth of biomarker investigation [16], adding new layers of information to the clinical and biological profiling of diseases [17–19]. Advancements in liquid chromatography and mass spectrometry (MS) instruments now enable almost full coverage of the protein-coding genome, and quantitation of even slight changes in protein expression [20, 21]. Furthermore, targeted MS techniques provide accurate and absolute quantitation of target analytes, making them suitable for both biomarker verification and clinical diagnostics [22, 23]. In our laboratory, we have developed a tissue proteomics biomarker discovery pipeline combining laser capture microdissection with high resolution MS analysis and label free quantitation for the analysis of breast carcinomas [24, 25], allowing us to not only establish a robust platform for biomarker discovery but also dissect the underlying biological mechanisms in epithelial tumors. Through this pipeline, we previously analyzed a cohort of snap frozen ER positive primary tumors and developed a 4-protein signature – comprising PDCD4, CNG, OCIAD1, and G3BP2 – predicting poor outcome to tamoxifen treatment with 86.7% sensitivity and 41.5% specificity [26]. However, the 4 proteins included in the classifier did not display extreme differential levels between tamoxifen outcome groups, suggesting that other factors may be involved in tamoxifen treatment outcome. In order to address this, we combined our initial MS cohorts and assessed which proteins manifested the highest change in expression between patient groups. The top candidates were selected based on significance after statistical evaluation of their abundance levels between patient groups, and their association to TTP was assessed in MS analyzed samples. Furthermore, IHC analysis of an independent cohort of tumors incorporated in a TMA provided further marker verification. In order to assess which pathways the top molecules were involved in, correlation and pathway analyses were performed. A schematic representation of the study's workflow is reported in Figure 1.

## RESULTS

### Analysis of MS sets

**Figure 2:**
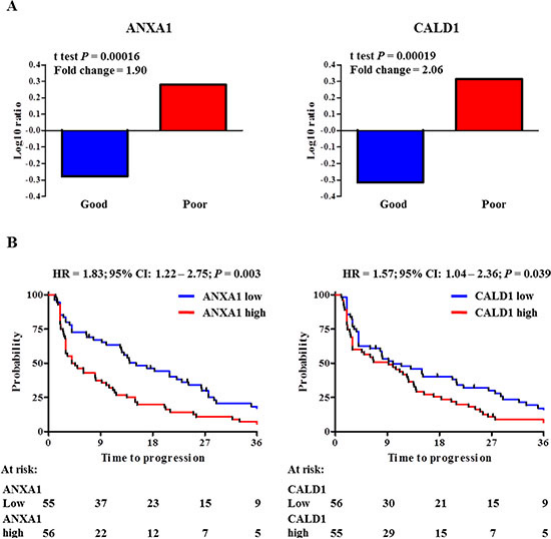
ANXA1 and CALD1 expression levels and survival analyses in MS cohorts Measurement of ANXA1 and CALD1 protein levels based on previously derived proteomic data. Panel A displays Log ratio bar charts show that both ANXA1 (*t* test *P* = 0.00016; Fold ratio = 1.90; left) and CALD1 (*t* test *P* = 0.00019; Fold ratio = 2.06; right) were highly differentially expressed in the poor outcome group. Stratification of patients according to median protein level showed that a significant difference was observed between ANXA1 (left) and CALD1 (right) protein levels (Panel B).

In order to assess which proteins showed the larger change in expression between good and poor outcome tamoxifen treatment groups, statistical analysis (i.e. *t* test) was performed on all 1,960 quantified proteins (Table S1) in our 112 patient MS cohort (Clinical and histo- pathological characteristics are reported in Table S2). Based on significance levels, ANXA1 (*t* test *P* < 0.001; fold change = 1.90) and CALD1 (*t* test *P* < 0.001; fold change = 2.06) proteins were selected as top candidates (Figure 2A and Figure S1). In addition to this, survival analysis was performed on ANXA1 and CALD1-stratified patients, with TTP as endpoint. A significant difference was observed between patients who displayed high levels (based on median expression) of ANXA1 (HR = 1.83; 95% CI: 1.22 – 2.75; *P = 0.003*) and CALD1 (HR = 1.57; 95% CI: 1.04 – 2.36; *P = 0.039*) proteins (Figure 2B). These data show that ANXA1 and CALD1 are positively associated to faster disease progression after tamoxifen treatment in ER positive recurrent breast cancer.

### Validation of ANXA1 and CALD1 as independent markers using immunohistochemistry

**Figure 3:**
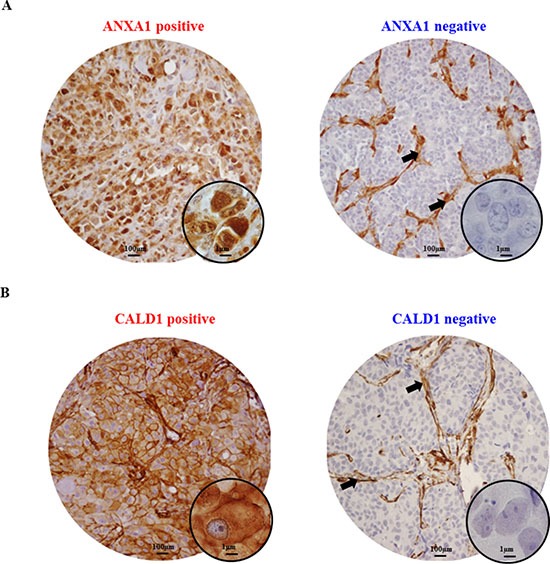
Immunohistochemical stainings of ANXA1 and CALD1 proteins Breast carcinomas included in the TMA displayed either ANXA1 positivity or negativity (**A**). Strong ANXA1 staining was found ubiquitously in stromal cells (black arrows) and was not taken into account in the survival analysis. CALD1 IHC staining was found at the membrane and cytoplasm of both carcinoma and stromal cells, but the latter was not taken into account for survival analyses (**B**).

**Figure 4:**
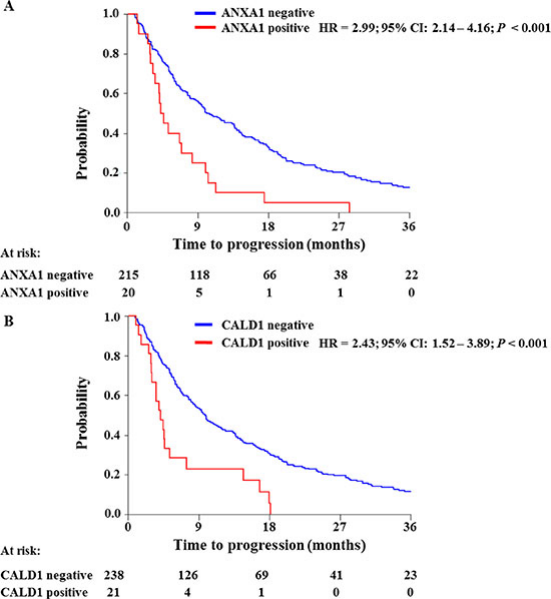
Survival analyses of ANXA1 and CALD1 association to TTP ANXA1 and CALD1 levels (i.e. negative/positive) were assessed by IHC and analyzed by Cox regression analysis and Log-rank test. Both ANXA1 (**A**) and CALD1 (**B**) levels showed significant association with short TTP in ER positive breast tumors.

In order to further confirm our findings, ANXA1 and CALD1 protein levels were assessed through IHC staining on an independent cohort of 408 FFPE tumor tissues derived from patients that received tamoxifen as first line therapy for recurrent breast cancer (Table S3). After filtering for missing values (Figure S2), a total of 20 out of 235 tumor tissues displayed ANXA1 positivity (Figure 3A). ANXA1 presence was significantly associated with shorter TTP in both univariate (HR = 2.99; 95% CI: 2.14 – 4.16; *P* < 0.001) and multivariate (HR = 1.82; 95% CI: 1.12 – 3.00; *P* = 0.016) Cox regression analyses (Table 1; Figure 4A). CALD1 positivity (Figure 3B) was observed in 21 out of 259 patients and Cox regression analysis showed a significant positive correlation with shorter TTP both in univariate (HR = 2.43; 95% CI: 1.52 – 3.89; *P* < 0.001) and multivariate (HR = 2.29; 95% CI: 1.40 – 3.75; *P* = 0.001) analyses (Table 2; Figure 4B). In addition to this, we analyzed whether the associations of ANXA1 and CALD1 with TTP were independent of each other in the subset of 235 tumor tissues for which both measurements were available. Multivariate Cox regression analysis showed that both ANXA1 (HR = 1.90; 95% CI: 1.16 – 3.10; *P* = 0.010) and CALD1 (HR = 2.29; 95% CI: 1.40 – 3.74; *P* = 0.001) were independently correlated to TTP (Table S4). Furthermore, in order to evaluate whether ANXA1 and CALD1 could be used in combination we merged IHC stainings into four categories: positive/positive, positive/negative, negative/positive, and negative/negative. ANXA1 and CALD1 staining only were observed in 17 and 16 tumor tissues, respectively, while only 4 tumors showed co-expression of the two markers. For all three positive categories (i.e. ANXA1 positive/CALD1 negative; ANXA1 negative/CALD1 positive; ANXA1 positive/CALD1 positive) an association with shorter TTP was found (Table S5, Figure S3). However, due to the low amount of tumors comprised in each category, further verification is needed to assess whether ANXA1 and CALD1 can be used in combination to effectively identify fast progressing breast carcinomas.

**Table 1:** Univariate and multivariate Cox regression analysis for the association of ANXA1 staining with TTP

		Univariate			Multivariate		
	n of patients	HR	95% CI	*P*	HR	95% CI	*P*
**ANXA1**							
**Negative**	272	1.00			1.00		
**Positive**	45	2.99	2.14 – 4.16	< 0.001	1.82	1.12 – 3.00	0.016
**Age**							
**≤ 55 years**	125	1.00			1.00		
**> 55 years**	192	0.59	0.47 – 0.75	< 0.001	0.56	0.42 – 0.75	< 0.001
**Disease-free survival**							
**≤ 12 months**	67	1.00			1.00		
**> 12 months**	250	0.69	0.52 – 0.91	0.008	0.72	0.51 – 1.01	0.057
**Dominant site of relapse**							
**Loco-regional**	43	1.00					
**Bone**	113	1.20	0.83 – 1.74	0.235			
**Visceral**	74	1.27	0.85 – 1.89	0.238			
**Bone and other**	87	1.25	0.85 – 1.84	0.258			
PgR*							
**Negative**	111	1.00			1.00		
**Positive**	204	0.51	0.40 – 0.64	< 0.001	0.71	0.52 – 0.97	0.034
Her2 status*							
**Negative**	201	1.00					
**Positive**	114	1.18	0.93 – 1.50	0.170			
Tumor differentiation*							
**Good**	46	1.00			1.00		
**Moderate**	150	1.45	1.01 – 2.06	0.042	1.15	0.79 – 1.67	0.456
**Poor**	118	1.95	1.35 – 2.82	< 0.001	1.24	0.82 – 1.89	0.311

* Missing data not reported.

Tumor differentiation was assessed through Bloom-Richardson scoring system.

Acronym: PgR: progesterone receptor.

**Table 2:** Univariate and multivariate Cox regression analysis for the association of CALD1 staining with TTP

		Univariate			Multivariate		
	n of patients	HR	95% CI	*P*	HR	95% CI	*P*
**CALD1**							
**Negative**	238	1.00			1.00		
**Positive**	21	2.43	1.52 – 3.89	< 0.001	2.29	1.40 – 3.75	0.001
Age*							
**≤ 55 years**	109	1.00			1.00		
**> 55 years**	150	0.64	0.50 – 0.83	0.001	0.55	0.41 – 0.73	< 0.001
**Disease-free survival**							
**≤ 12 months**	45	1.00			1.00		
**> 12 months**	214	0.72	0.52 – 1.00	0.052	0.76	0.52 – 1.11	0.158
**Dominant site of relapse**							
**Loco-regional**	29	1.00					
**Bone**	103	1.36	0.88 – 2.12	0.166			
**Visceral**	58	1.34	0.83 – 2.16	0.229			
**Bone and other**	69	1.47	0.92 – 2.34	0.105			
**PgR**							
**Negative**	65	1.00			1.00		
**Positive**	194	0.73	0.54 – 0.97	0.031	0.70	0.50 – 0.96	0.029
Her2 status**							
**Negative**	158	1.00					
**Positive**	79	1.18	0.91 – 1.54	0.221			
Tumor differentiation**							
**Good**	37	1.00					
**Moderate**	186	1.21	0.82 – 1.77	0.333			
**Poor**	35	1.5	1.00 – 2.26	0.051			

* Age was assessed at start of tamoxifen therapy.

** Missing data not reported.

Tumor differentiation was assessed through Bloom-Richardson scoring system.

Acronym: PgR: progesterone receptor

Logistic regression analysis showed a significant association of ANXA1 staining to clinical benefit to tamoxifen therapy in univariate analysis (Odds ratio [OR] = 0.22; 95% CI: 0.11 to 0.45; *P* < 0.001) and a borderline association after correction for traditional predictive factors (OR = 0.38; 95% CI: 0.15 to 1.01; *P =* 0.052; Table S6). The association of ANXA1 with objective response was found significant only in the univariate analysis (OR = 0.20; 95% CI: 0.46 to 0.84; *P =* 0.028; Table S7). A significant association was found between CALD1 staining and no clinical benefit both in univariate (OR = 0.21; 95% CI: 0.08 to 0.57; *P* = 0.002) and multivariate (OR = 0.21; 95% CI: 0.08 to 0.56; *P* = 0.002) logistic regression analysis (Table S8). No association was found between CALD1 staining and objective response (Table S9). Due to the fact that only CALD1 showed significant association with the type of response, while ANXA1 displayed only borderline significance, combination of ANXA1 and CALD1 stainings was not performed using tumor response as the endpoint of tamoxifen therapy. Overall, these data suggest a significant relationship between ANXA1 and CALD1 positivity and early tumor progression after tamoxifen treatment for recurrent ER positive breast cancer.

### Pathway analysis of ANXA1 and CALD1

**Figure 5:**
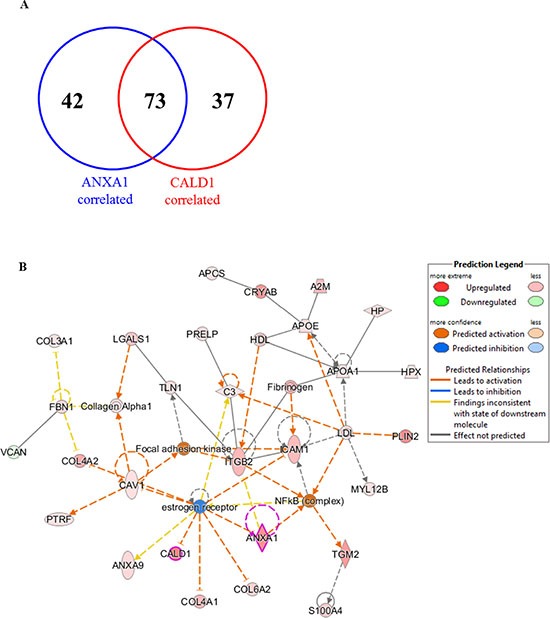
Interaction pathways derived from proteins correlated to ANXA1 and CALD1 Proteins associated with both CALD1 and ANXA1 were combined into one list (**A**) and submitted to IPA. Molecular network analysis showed that both ANXA1 and CALD1 were involved in downregulation of ER and activation of the NFκB pathway (**B**). Expressed molecules in the pathway were: A2M (alpha-2-macroglobulin), ANXA1 (Annexin-A1), ANXA9 (Annexin-A9), APCS (Amyloid P component), APOA1 (apolipoprotein-A1), C3 (Complement C3), CALD1 (Caldesmon), CAV1 (Caveolin-1), COL3A1 (collagen type III alpha 1), COL4A1 (collagen type IV alpha 1), COL4A2 (collagen type IV alpha 2), COL6A2 (collagen type VI alpha 2), CRYAB (Crystallin alpha B), FBN1 (Fibrillin-1), HP (Haptoglobin), HPX (Hemopexin), ICAM1 (Intercellular adhesion molecule 1), ITGB2 (Integrin beta 2), LGALS1 (Lectin galactoside-binding soluble 1), MYL12B (Myosin light chain 12B), PLIN2 (Perilipin-2), PRELP (Proline/arginine-rich end Leucine-rich repeat protein), PTFR (Polymerase I and transcript release factor), S100A4 (Calcium binding protein S100A4), TGM2 (Transglutaminase 2), TLN1 (Talin-1), and VCAN (Versican).

As ANXA1 and CALD1 showed to be highly expressed in the poor outcome patient group, Pearson correlation analysis of abundance levels of all proteins in the MS dataset to ANXA1 and CALD1 expression was performed. After selection of only highly correlated proteins, correlation analysis yielded a total of 115 (e.g. SERPINH1) and 110 (e.g. Vinculin) proteins correlated to ANXA1 and CALD1, respectively. Of these, 73 (i.e. more than 60% in both lists) correlated with both ANXA1 and CALD1 levels (Tables S10 and S11; Figure 5A), suggesting that these proteins presented a shared biology. In the light of this we merged the two correlated protein lists and performed pathway analysis of ANXA1 and CALD1 and their associated proteins through IPA software. Canonical pathway analysis showed activation of acute phase response signaling (Z-score = 3.00; Fisher *P* = 9.59E– 10; Table S12), pointing out that molecules involved in inflammation might be correlated to tamoxifen poor outcome. Upstream analysis showed that ER signaling was downregulated (Z-score = −2.111; Fisher's *P* = 1.39E–07; Table S13), suggesting a link between ANXA1 and CALD1 related proteins in the disruption of ER signaling. Molecular interaction analysis indicated that both CALD1 and ANXA1 were comprised in a network along with proteins involved in cellular movement and immune response (Focus molecules: 28; *P*-score = 48; Figure 5B). This network comprised proteins belonging to the extracellular matrix (e.g. APOE; COL4A2) and proteins involved in cell movement (e.g. MYL12B) and cell adhesion (e.g. ICAM1). Molecule activity prediction based on proteins Log ratios pointed out that molecules in the network were not only involved in ER down-regulation, but were also associated with the activation of the focal adhesion kinase and NFκB pathways. Overall, these data suggest that ANXA1 and CALD1, along with their correlated proteins, are associated with down-regulation of ER signaling and the activation of inflammation response mechanisms. However, further verification in breast cancer model systems are required to confirm these hypotheses.

## DISCUSSION

Resistance to tamoxifen is still a major cause of death in patients with ER positive recurrent breast cancer [27]. The advanced state of current proteomic technologies allows profiling of biological samples for discovery of disease biomarkers [28]. Indeed also in recurrent ER positive breast cancer, using our dedicated tissue proteomics workflow, we developed and validated a 4-protein signature predictive of tamoxifen treatment outcome [26]. Despite the fact that our predictor is capable of discriminating patient groups displaying good and poor outcome to tamoxifen therapy, the 4 signature proteins alone are unlikely capable of addressing the full extent of resistance mechanisms. Out of a panel of 1,960 proteins, ANXA1 and CALD1 constituted the top 2 significant proteins and were shown to be overexpressed in the poor outcome group. Furthermore ANXA and CALD1 have already been described as cell migration and markers of an epithelial-to-mesenchymal (EMT) –like phenotype in ER negative breast cancer cell line models [29, 30], though their role in ER positive tumors still needs to be functionally elucidated.

In order to confirm our MS findings, we performed IHC stainings of both ANXA1 and CALD1 in an independent cohort of FFPE ER positive tumor tissues captured in a TMA to assess the clinical relevance of these markers. Our regression analyses showed that not only ANXA1 (HR = 1.82) and CALD1 (HR = 2.40) stainings were significantly associated with shorter TTP independently of traditional predictive factors, but also contributed in stratifying patient groups independently of each other. Combination of IHC stainings suggested that ANXA1 and CALD1 could be used in concert to discriminate patients who would suffer from fast disease progression after first line tamoxifen, however the relatively small size of these groups implies that further verification in a larger patient cohort is necessary. Though association of CALD1 to breast cancer therapy outcome has been poorly assessed so far, ANXA1 expression was previously associated to BRCA1/2 mutation carriers and prediction of high mortality risk in Her2^+^ patients [30]. Our data might suggest an additional role of ANXA1 and CALD1 in disease progression after tamoxifen therapy of ER positive recurrent breast cancers.

In order to better investigate which proteins and molecular pathways were connected to ANXA1 and CALD1, we performed pathway analysis on a cohort of ER positive tumors which were previously analyzed by high resolution MS. Proteins that were found to be correlated to ANXA1 and CALD1 levels were associated not only with inflammatory response (canonical pathway analysis), but also with activation of the NFκB pathway, downregulation of ER signaling and focal adhesion (molecule activity prediction). Similar findings were reported before as ANXA1 has been shown to be associated with increased metastatic potential in MCF-7 breast cancer cell lines following downregulation of ER and the expression of basal markers (e.g. vinculin) [31]. Moreover, studies on breast cancer cell line models have shown that constitutive activation of the NFκB pathway leads to downregulation of ER signaling [32], which can have a prominent role in tamoxifen resistance, if not in a generalized anti-hormonal therapy unresponsiveness. In another study elucidating the role of ANXA1 in relation to NFκB activation, it was shown that its interaction with the IKK complex led to the constitutive activation of this pathway, promoting metastasis and decreasing survival in an intracardiac metastatic model [33]. The constitutive activation of NFκB signaling in cancer cells favors not only cell survival, but also the acquisition of a more malignant phenotype [34]. ANXA1 was also associated with acquisition of an EMT-like phenotype in ER negative breast cancer cell line models through activation of TGFβ signaling [28]. Although pathway analysis did not show a direct activation of the TGFβ pathway, cross-talk between TGFβ and NFκB has already been described in various forms of cancer, in which one pathway modulates the other via activation of binding proteins [35] or of micro-RNAs [36]. In contrast, it has been shown that expression of ANXA1 was related to inhibition of NFκB in pancreatic and colon cancer cell lines [37], while in Adriamycin-resistant bladder cancer, ANXA1 has been reported to be downregulated [38]. In the light of this, ANXA1 may be associated with the constitutive activation of NFκB signaling in a tissue-specific manner, which would on one side lead tumor cells to become estrogen-independent and on the other side promote a more aggressive and mesenchymal-like phenotype, though further studies need to be performed in order to confirm this function. Nonetheless, a possible novel treatment option for ANXA1 positive, tamoxifen resistant tumors would consist of blocking antibodies, which have already shown efficacy in inhibiting migration and invasion rates in pancreatic carcinoma cells [39].

CALD1 is an actin-, calmodulin- and myosin-binding protein that has been described as a cell motility suppressor by stabilizing stress fibers through F-actin binding [40, 41]. Despite its role in downregulating cell motility by cytoskeletal rearrangements, CALD1 has also been described as a key component in TGFβ-driven EMT via its overexpression [42]. In addition to this, downregulation of ER in MCF7 cells has been linked to the upregulation of CALD1, concomitantly with the acquisition of a new phenotype that encompasses increased growth rates, loss of cell-to-cell adhesion and a redistribution of the cytoskeletal components, resulting in increased motility [31]. In addition to this, CALD1 interaction with cGMP-dependent protein kinase Iβ has been shown to regulate cell invasion and migration in breast cancer cell lines [29]. Similar to ANXA1, also CALD1 has been shown to display opposite roles in cancer and invasion, since its expression has been associated to reduced cell invasion in colon cancer cell lines [43]. In this perspective, CALD1 may be another key effector in cytoskeletal rearrangements and the acquisition of a rapid spreading tumor phenotype in breast tissue, while countering those effects in other tissue types.

As both ANXA1 and CALD1 have been reported to be expressed in basal-type breast cancers, which are characterized by downregulation of ER and its responsive genes [44], and the fact that only a minority of the ER positive tumors that were captured in the TMA displayed expression of these markers, it is possible that these proteins may enable further stratification of ER positive breast malignancies. In the perspective of tamoxifen resistance, ANXA1 and CALD1 may be involved in the activation of the NFκB pathway, which would promote cell survival by blocking intrinsic (mitochondrial-mediated) and extrinsic (death receptors-mediated) apoptotic signals and render cancer cells independent of estrogens. In addition to this, the acquisition of a rapidly spreading and fast growing tumor cell phenotype would result in a faster tumor progression, and probably an estrogen-independent phenotype.

We have here shown that ANXA1 and CALD1 are associated with tamoxifen therapy clinical outcome in recurrent ER positive breast cancer. Expression of these proteins not only correlates with shorter TTP independently of traditional predictive factors, but these markers also contributed independently of one another. In other words, ANXA1 and CALD1, alone or in combination, are able to identify groups of patients that would less likely benefit from tamoxifen therapy. In addition to this, pathway analysis suggested that ANXA1 and CALD1 are likely linked to the downregulation of ER signaling and acquisition of a more malignant phenotype with EMT-like features. Blocking such pathways would probably constitute an effective additional or substitutive therapy in patients expressing such markers, however further functional studies should be performed in order to determine causal effects of these proteins in these signaling cascades.

## MATERIALS AND METHODS

### MS dataset

A total of 112 ER positive tissues were analyzed by high resolution MS after LCM-based breast carcinoma cell enrichment, as previously described [26]. MaxQuant (version 1.2.2.5; search engine: Andromeda) [45, 46] results of Orbitrap. RAW data deposited in ProteomeXchange via PRIDE repository (dataset identifiers: PXD000484 and PXD000485) [47] from previously described sets [26] were imported in Microsoft Excel and normalized for inter-batch effects using ComBat algorithm in R environment [48] allowing 10 minimum observations across all samples. Outcome to tamoxifen treatment was defined based on TTP, with a 6 months cutoff: patient whose tumors progressed within (≤) 6 months after start of therapy were defined as poor outcome, while progression after (>) 6 months was defined as good outcome. This set comprised 67 good and 47 poor outcome patients, respectively. Protein differential expression between patient groups was tested by *t* test (two sided test, unequal variances assumed) in Microsoft Excel. Top candidates were selected based on *t* test *P* and their relation to TTP was confirmed by survival analysis through survival analysis of patients stratified according to median level of protein expression.

### TMA dataset

A total of 447 FFPE tissues collected from Erasmus MC and regional hospitals were incorporated in a TMA. For statistical analysis only ER positive tumors from patients who did not receive any adjuvant hormonal therapy were included. Furthermore, patients who showed no tumors after histological revision or manifested disease progression before (≤) 3 weeks after start of therapy were excluded. In addition, tumors that were comprised in the MS sets were also excluded. This led to the inclusion of a total of 408 ER positive tumors, of which response data were collected according to the standard International Union Against Cancer criteria [49]. Eleven (2.70%) and 51 (12.50%) patients showed complete remission (CR) and partial remission (PR), respectively. Two hundred and five patients showed no change (NC) of disease, of whom 35 (8.58%) displayed NC for less (≤) than 6 months, while 170 (41.66%) showed NC for longer (>) than 6 months (defined as stable disease [SD]). A total of 141 (34.56%) patients displayed progressive disease (PD). Clinical benefit was defined as CR + PR + SD patients (*n* = 232; 57%), while objective response was defined as CR + PR only (*n* = 62; 15%). This retrospective study used coded primary tumor tissues, in accordance with the Code of Conduct of the Federation of Medical Scientific Societies in the Netherlands (http://www.federa.org/codes-conduct). Reporting Recommendations for Tumor Marker Prognostic Studies were followed where possible [50].

### Tissue micro-array

TMA was prepared using an ATA 27 (Beecher Instruments, Sun Prairie, WI, USA). 447 paraffin-embedded primary, ER positive breast cancer tissues derived from patients who received tamoxifen as first-line therapy for recurrent disease were used to prepare the TMA. Tissue cores of 0.6 mm were taken from each tissue paraffin block and transferred in triplicate into a TMA recipient block. For each tumor tissue sample, three different areas of the tumor were taken as biological replicates. TMA slides were digitalized and analyzed using Slidepath software (Leica Microsystems, Solms, Germany).

### Immunohistochemistry

Five μm sections of FFPE tissues captured on the TMA were incubated at 60°C and washed in xylene (3 × 5 min) for de-paraffination. Washings with decreasing concentrations of ethanol were used to re-hydrate tissues as follows: 100% ethanol (1 × 5 min, 2 × 2 min), 70% ethanol (1 × 2 min), 50% ethanol (1 × 2 min), distilled water (1 × 2 min). Incubation with DAKO (Agilent Technologies Inc, Glostrup, Denmark) antigen retrieval solution diluted 1:10 in MilliQ water was performed at 95°C for 40 min. Slides were then cooled down to room temperature and washed with PBS (3 × 5 min). Slides were first incubated with 0.003% H_2_O_2_ in PBS to block endogenous peroxidase (10 min) and subsequently with blocking solution consisting of 5% BSA in PBS for 30 min. Anti-ANXA1 (Clone ID: 29/Annexin I; Transduction Laboratories, Lexington, KY, USA) and anti-CALD1 (Clone ID: TD107; Enzo Life Sciences Inc., Farmingdale, NY, USA) mouse monoclonal primary antibodies were diluted 1:2000 (ANXA1) and 1:400 (CALD1), respectively, in DAKO Antibody Diluent, and slides were incubated for 1 h at room temperature. Slides were then washed with PBS, and DAKO Envision^®^ secondary antibody (labeled polymer HRP -Mouse, 200 μl per slide) solution was added to each slide and incubated for 45 min at room temperature. A washing cycle with PBS was performed for 5 min and a 1:15 solution of DAB+ chromogen in DAB+ substrate buffer was added, following incubation in the dark for 10 min. Slides were then washed in tap water for 5 min, stained with hematoxylin/eosin for 1 min each and dehydrated again through sequential washings in 50%–70%–100% ethanol and xylene of 5 min each. Cover glasses were mounted with Pertex and slides were left to dry. ANXA1 and CALD1 stained tissue sections were scored only for quantity of stained carcinoma cells due to the fact that all stained tumors displayed strong staining intensity. Scoring was performed by an experienced researcher in a blind manner, and triplicate scores were assessed and validated by a second experienced researcher, who was extensively trained by a specialized breast pathologist. Fibroblasts, endothelial cells and leukocytes displayed strong ubiquitous ANXA1 and CALD1 stainings as well, while adipocytes only stained for ANXA1 (Figure S4A–S4B). Only staining in breast carcinoma cells were taken into account for further analyses. Slides were digitalized and analyzed using Slidepath software (Leica Microsystems, Solms, Germany).

### Statistical analysis of IHC staining results

Staining scores of TMA incorporated tissues were filtered for missing data, stringently excluding cores for which triplicate measurement was not available (e.g. due to loss of core during staining procedure or not enough [< 35] carcinoma cells observed in at least one core), leading to a set of 317 tissue samples for ANXA1, a list of 259 tissues for CALD1, and a list of 235 tissues for both ANXA1 and CALD1 (Figure S2). Due to the fact that tumor tissues displayed high quantities of stained breast carcinoma cells, both ANXA1 and CALD1 were scored as either absent or present. Association of ANXA1 and CALD1 stainings with TTP or response data were tested by Cox and logistic regression, respectively. Patient age, disease free interval, dominant site of relapse, progesterone receptor (PgR) positivity, HER2 overexpression, and degree of tumor differentiation (Bloom-Richardson) were included in all regression analyses. With the exception of disease-free survival (to correct for prognosis), variables that did not display any significant association with TTP or any response criteria were excluded from the multivariate regression models. Cox regression, logistic regression, HRs, OR, and 95% CIs were calculated in Stata (v 13.1; Stata Corp, College Station, TX, USA).

### Extraction of proteomics data and pathway analysis

ANXA1 and CALD1 levels were correlated with the rest of the quantified dataset through Pearson correlation. The distribution of correlation coefficients was analyzed by Z-score; cutoffs were selected according to the following formula:

Z-score cutoffs=mean±1.96*(standard deviation)

Z-score transformed correlation coefficients that fell below (< mean – 1.96 * [standard deviation]) or above (> mean + 1.96 * [standard deviation]) the cutoffs were selected as highly correlated proteins. IPA^®^ (IPA, Qiagen, Redwood City, www.qiagen.com/ingenuity) was performed selecting Uniprot identifiers and Log10 ratios as input data (no ratio cutoff was selected). Pathway analysis was run using Ingenuity Knowledge Base as reference database. Analysis was run with the following options enabled: *homo sapiens* was selected as species; human tissues and breast cancer cell lines were selected as protein expression sites; Mutation and Data Sources options were kept as default (i.e. All). Interaction networks were generated including endogenous chemicals and maintaining other options as default (i.e. number of Molecules per network: 35; number of Networks per analysis: 25). Networks that showed the highest *P*-score (i.e. *P*-score = −Log10 [Fisher's *P*]) were subjected to Molecule Activity Predictor in IPA.

## SUPPLEMENTARY FIGURES AND TABLES








