# Supplementary material for: Annexin-A1 and caldesmon are associated with resistance to tamoxifen in estrogen receptor positive recurrent breast cancer
Source: Oncotarget. 2015 Dec 9;7(3):3098–110. doi: 10.18632/oncotarget.6521 (PMC4823093; doi:10.18632/oncotarget.6521)
Supplement: Supplementary file 1 [file oncotarget-07-3098-s001.pdf]

Annexin-A1 and caldesmon are associated with resistance to tamoxifen in estrogen receptor positive recurrent breast cancer

Supplementary Materials

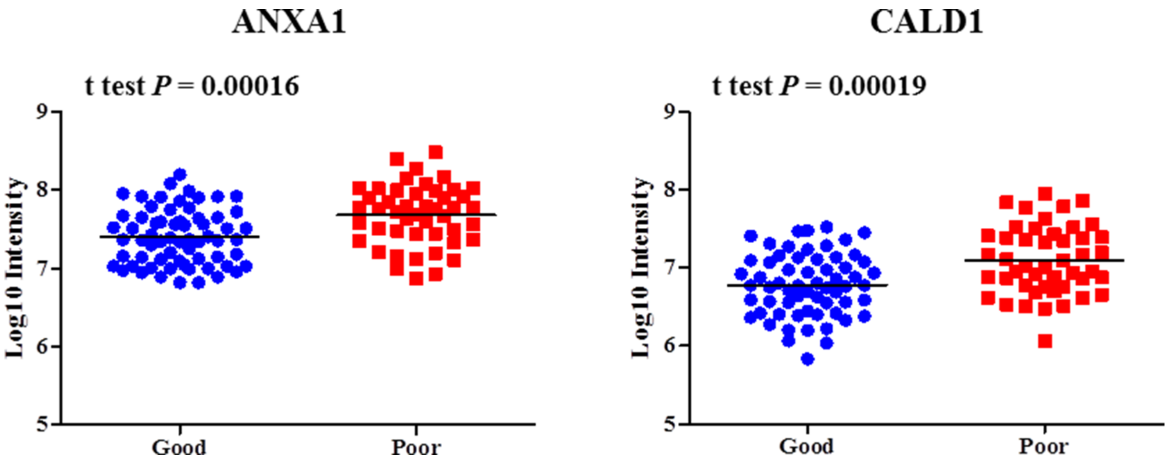

Supplementary Figure S1: Scatter dot plots of ANXA1 and CALD1 levels in the MS sets.

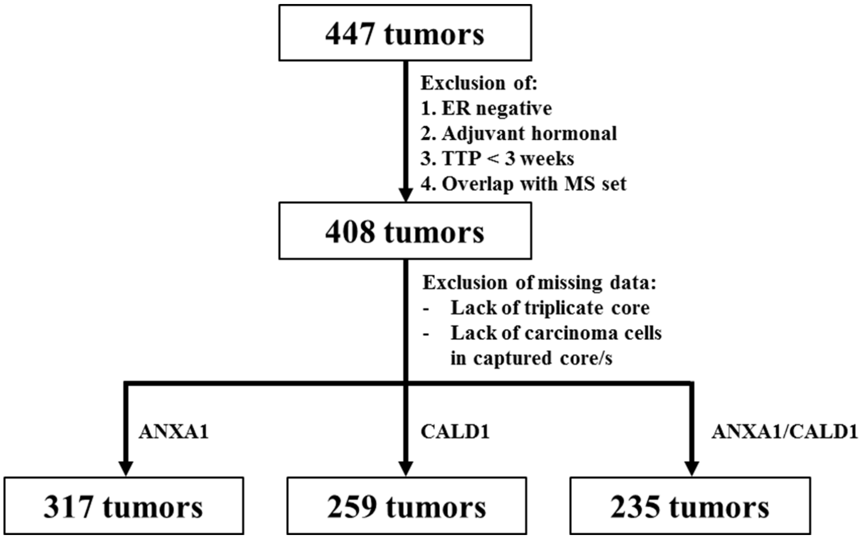

Supplementary Figure S2: Filtering scheme of TMA incorporated tissues. Of all tissues included in the TMA, only ER positive tumors from patients that were treatment naïve and displayed TTP longer than 3 weeks were included in the statistical analysis. Missing data were the result of lost tissue cores during the cutting and staining procedure or poor staining quality, which prevented triplicate evaluation of tumor tissues. *Acronyms: ER: estrogen receptor; TTP: time to progression.

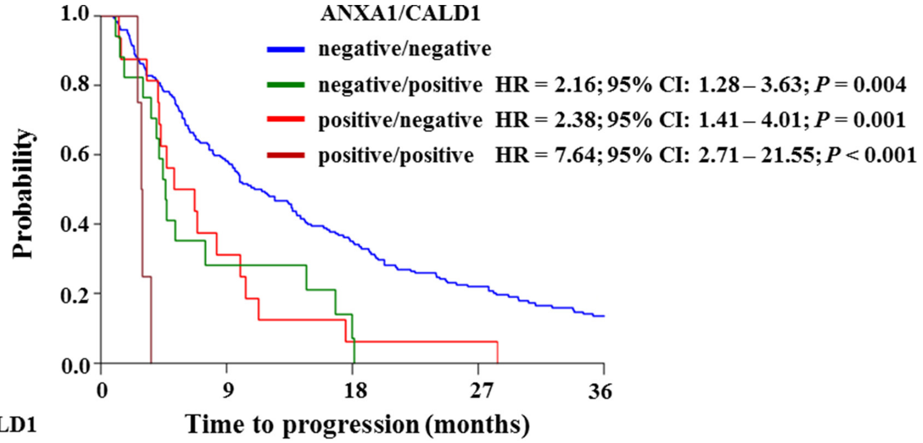

At risk: ANXA1/CALD1	0	9	18	27	36
negative/negative	198	114	35	38	22
negative/positive	17	4	1	0	0
positive/negative	16	5	1	1	0
positive/positive	4	0	0	0	0

Supplementary Figure S3: Survival analysis of combined ANXA1 and CALD1 stainings. ANXA1 and CALD1 stainings were combined into 4 categories: positive/positive, positive/negative, negative/positive and negative/negative and survival analysis was performed to assess their relation to TTP.

A

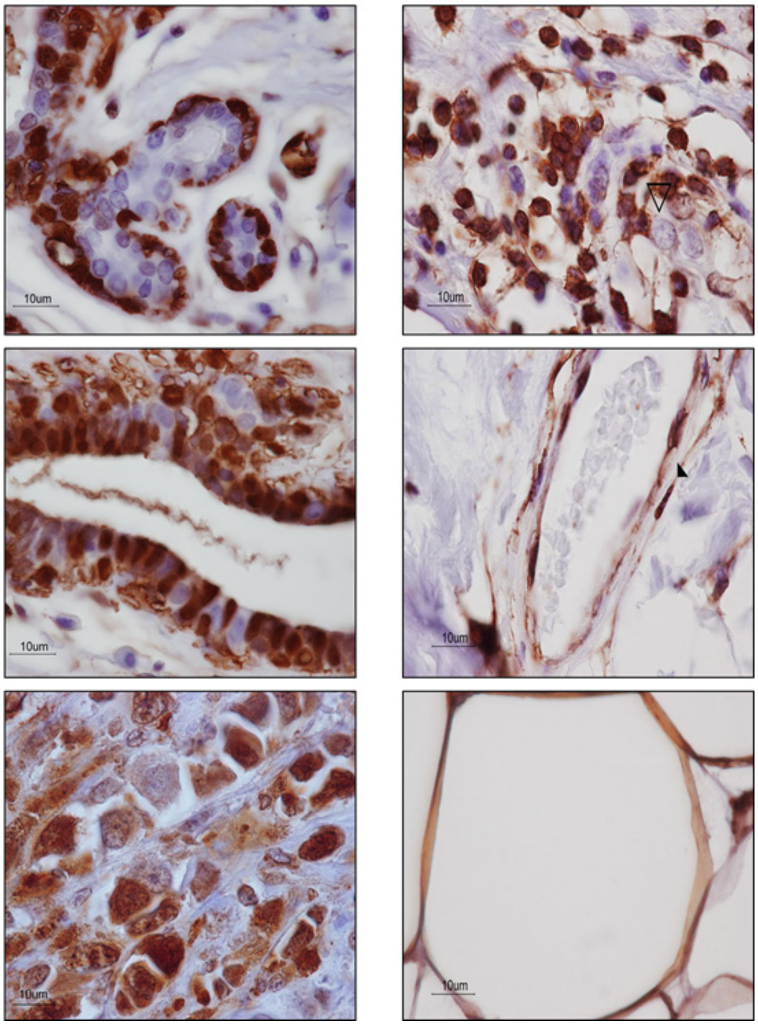

B

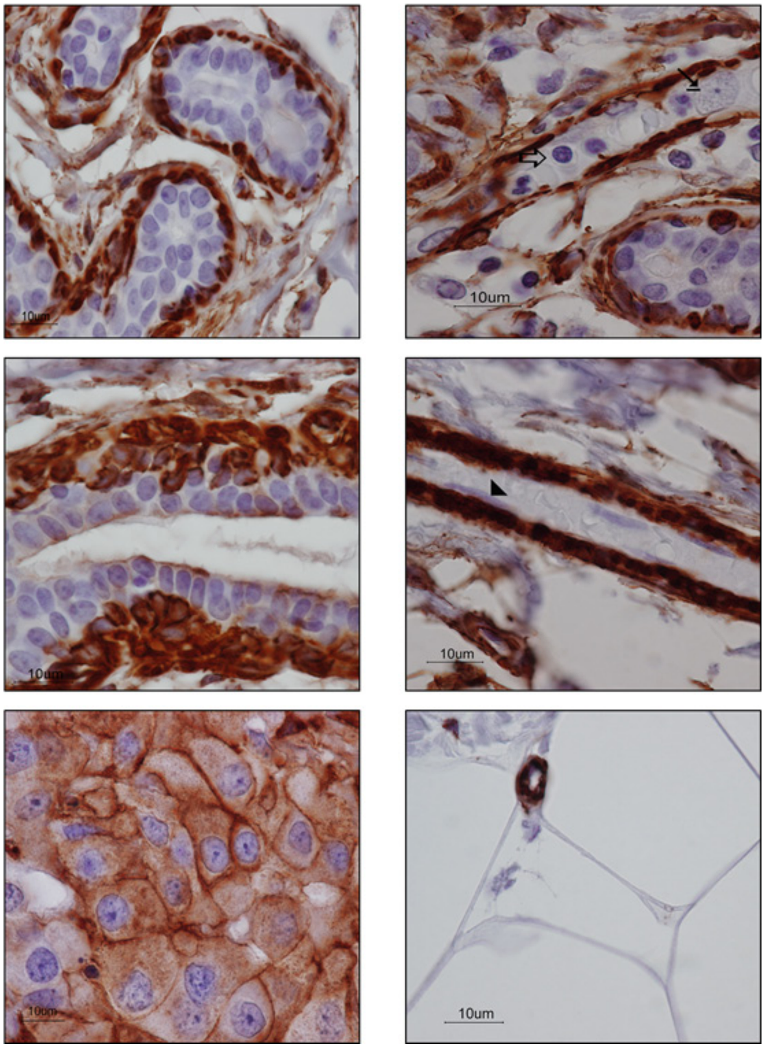

Supplementary Figure S4: Comparative of IHC stainings of ANXA1 and CALD1. Panel (**A**) and (**B**) display ANXA1 and CALD1 stainings respectively. Stainings are displayed in normal breast acini (top left), normal breast ducts (middle left), breast carcinoma cells (bottom left), leucocytes (top right), blood vessels and adjacent fibroblasts (middle left) and adipocytes (bottom left). Triangles indicate (negative) tumor cells; arrows and empty arrows show tumor cells and leucocytes respectively; arrow tip shows (negative) endothelial cells.

Supplementary Table S2: Clinical information of patients included in MS sets

	Patients (%)
**All patients**	112 (100)
**Age***	
**≤ 55 years**	28 (25)
**> 55 years**	84 (75)
**Menopausal status***	
**Premenopausal**	21 (19)
**Postmenopausal**	91 (81)
**Tumor size**	
**T1 (≤ 2 cm)**	37 (33)
**T2 (2-5 cm) + Tx**	68 (61)
**T3 (> 5 cm) + T4**	7 (6)
**Tumor differentiation****	
**Good/Moderate**	50 (45)
**Poor**	49 (43)
**Unknown**	13 (12)
**Disease free interval**	
**≤ 12 months**	33 (29)
**> 12 months**	79 (71)
**PgR†**	
**Negative**	37 (33)
**Positive**	72 (64)
**Involved lymph nodes**	
**0**	61 (55)
**≥ 1**	43 (38)
**unknown**	8 (7)
**Dominant site of relapse**	
**Loco-regional**	12 (11)
**Bone**	44 (39)
**Visceral**	28 (25)
**Bone and other**	23 (21)
**Unknown**	5 (4)

*Age and menopausal status were assessed at start of tamoxifen therapy.
** Histopathological characteristics were evaluated by local pathologists, according to standard clinical practice at time of sample collection.
†Missing data not reported.
Acronym: PgR: progesterone receptor

Supplementary Table S3: Clinical information of patients included in tissue micro-array

	Patients (%)
**All patients**	**408 (100)**
**Age***	
**≤55 years**	164 (40)
**>55 years**	244 (60)
**Menopausal status***	
**Premenopausal**	106 (26)
**Postmenopausal**	302 (74)
**Tumor size**	
**T1 (≤2 cm)**	153 (37)
**T2 (2-5 cm) + Tx**	211 (52)
**T3 (>5 cm) + T4**	44 (11)
**Tumor differentiation****	
**Good/Moderate**	266 (65)
**Poor**	139 (34)
**Unknown**	3 (1)
**Involved lymph nodes†**	
**0**	132 (32)
**≥1**	266 (65)
**Disease free interval**	
**≤12 months**	85 (21)
**>12 months**	323 (79)
**Dominant site of relapse**	
**Loco-regional**	51 (12)
**Bone**	152 (37)
**Visceral**	93 (23)
**Bone and other**	112 (28)
**PgR****	
**Negative**	150 (37)
**Positive**	253 (62)

*Age and menopausal status were assessed at start of tamoxifen therapy.
**Tumor differentiation was evaluated through Bloom-Richardson grading system
†Missing data not reported
Acronyms: PgR: progesterone receptor

Supplementary Table S4: Univariate and multivariate analysis for the association of ANXA1 and CALD1 stainings with TTP

		Univariate			Multivariate		
	n of patients	HR	95% CI	*P*	HR	95% CI	*P*
**CALD1***							
**Negative**	214	1.00			1.00		
**Positive**	21	2.51	1.60 – 4.12	< 0.001	2.29	1.40 – 3.74	0.001
**ANXA1***							
**Negative**	215	1.00			1.00		
**Positive**	20	2.34	1.40 – 3.74	< 0.001	1.90	0.16 – 3.10	0.010
**Age****							
**≤ 55 years**	97	1.00			1.00		
**> 55 years**	138	0.61	0.47 – 0.80	< 0.001	0.58	0.44 – 0.77	< 0.001
**Disease-free survival**							
**≤ 12 months**	40	1.00			1.00		
**> 12 months**	195	0.74	0.52 – 1.04	0.087	0.73	0.50 – 1.07	0.106
**Dominant site of relapse**							
**Loco-regional**	28	1.00					
**Bone**	94	1.38	0.87 – 2.16	0.166			
**Visceral**	52	1.37	0.84 – 2.24	0.209			
**Bone and other**	61	1.43	0.87 – 2.31	0.142			
**PgR**							
**Negative**	57	1.00			1.00		
**Positive**	178	0.69	0.51 – 0.95	0.022	0.76	0.54 – 1.05	0.099
**Her2 status***							
**Negative**	153	1.00					
**Positive**	81	1.20	0.91 – 1.59	0.196			
**Tumor differentiation***							
**Good**	33	1.00					
**Moderate**	123	1.24	0.83 – 1.87	0.291			
**Poor**	78	1.50	0.97 – 2.31	0.069			

*Age was assessed at start of tamoxifen therapy.
**Missing data not reported
Tumor differentiation was assessed through Bloom-Richardson scoring system.
Acronym: PgR: progesterone receptor

Supplementary Table S5: Univariate and multivariate Cox regression analysis for the association of ANXA1/CALD1 stainings with TTP

		Univariate			Multivariate		
	**n of patients**	**HR**	**95% CI**	***P***	**HR**	**95% CI**	***P***
**ANXA1/CALD1**							
**Negative/Negative**	198	1.00			1.00		
**Negative/Positive**	17	2.16	1.28 – 3.63	0.004	1.73	1.01 – 2.95	0.045
**Positive/Negative**	16	2.38	1.41 – 4.01	0.001	2.06	1.20 – 3.55	0.009
**Positive/Positive**	4	7.64	2.71 – 21.55	< 0.001	7.10	2.42 – 20.85	< 0.001
**Age***							
**≤ 55 years**	97	1.00			1.00		
**> 55 years**	138	0.61	0.47 – 0.08	< 0.001	0.57	0.43 – 0.76	< 0.001
**Disease-free survival**							
**≤ 12 months**	40	1.00			1.00		
**> 12 months**	195	0.74	0.52 – 1.04	0.087	0.71	0.49 – 1.04	0.076
**Dominant site of relapse**							
**Loco-regional**	28	1.00					
**Bone**	94	1.38	0.87 – 2.16	0.166			
**Visceral**	52	1.37	0.84 – 2.24	0.209			
**Bone and other**	61	1.43	0.89 – 2.32	0.142			
**PgR**							
**Negative**	57	1.00			1.00		
**Positive**	178	0.69	0.51 – 0.95	0.022	0.77	0.55 – 1.08	0.126
**Her2 status****							
**Negative**	153	1.00					
**Positive**	81	1.20	0.91 – 1.59	0.196			
**Tumor differentiation****							
**Good**	33	1.00					
**Moderate**	123	1.24	0.83 – 1.87	0.291			
**Poor**	78	1.50	0.97 – 2.31	0.069			

*Age was assessed at start of tamoxifen therapy.
**Missing data not reported.
Tumor differentiation was assessed through Bloom-Richardson scoring system.
Acronym: PgR: progesterone receptor

Supplementary Table S6: Univariate and multivariate analysis for the association of ANXA1 staining with clinical benefit

		Univariate			Multivariate		
	n of patients	HR	95% CI	*P*	HR	95% CI	*P*
**ANXA1**							
**Negative**	272	1.00			1.00		
**Positive**	45	0.22	0.11 – 0.45	< 0.001	0.38	1.15 – 1.01	0.052
**Age***							
**≤ 55 years**	125	1.00			1.00		
**> 55 years**	192	1.71	1.08 – 2.70	1.021	2.14	1.22 – 3.75	0.008
**Disease-free survival**							
**≤ 12 months**	67	1.00			1.00		
**> 12 months**	250	1.85	1.07 – 3.19	0.027	1.37	0.68 – 2.76	0.384
**Dominant site of relapse**							
**Loco-regional**	43	1.00					
**Bone**	113	0.63	0.30 – 1.32	0.221			
**Visceral**	74	0.43	0.20 – 0.95	0.037			
**Bone and other**	87	0.71	0.33 – 1.55	0.397			
**PgR**							
**Negative**	111	1.00			1.00		
**Positive**	204	3.16	1.96 – 5.12	<0.001	1.67	0.89 – 3.12	0.109
**Her2 status***							
**Negative**	201	1.00					
**Positive**	114	0.75	0.47 – 1.91	0.224			
**Tumor differentiation***							
**Good**	46	1.00			1.00		
**Moderate**	150	0.59	0.29 – 1.21	0.152	0.77	0.34 – 1.71	0.521
**Poor**	118	0.34	0.16 – 2.72	0.005	0.68	0.29 – 1.60	0.378

*Age was assessed at start of tamoxifen therapy.

**Missing data not reported

Tumor differentiation was assessed through Bloom-Richardson scoring system.

Acronym: PgR: progesterone receptor

Supplementary Table S7: Univariate and multivariate analysis for the association of ANXA1 staining with objective response

		Univariate			Multivariate		
	**n of patients**	**HR**	**95% CI**	***P***	**Odds ratio**	**95% CI**	***P***
**ANXA1**							
**Negative**	272	1.00			1.00		
**Positive**	45	0.20	0.46 – 0.84	0.028	0.22	0.03 – 1.81	0.161
**Age***							
**≤ 55 years**	125	1.00			1.00		
**> 55 years**	192	2.09	1.08 – 4.02	0.028	2.62	1.25 – 5.50	0.010
**Disease-free survival**							
**≤ 12 months**	67	1.00			1.00		
**> 12 months**	250	1.66	0.74 – 3.72	0.215	1.90	0.75 – 4.77	0.173
**Dominant site of relapse**							
**Loco-regional**	43	1.00			1.00		
**Bone**	113	0.22	0.09 – 0.53	0.001	0.13	0.05 – 0.36	< 0.001
**Visceral**	74	0.29	0.12 – 0.73	0.008	0.17	0.05 – 0.49	0.001
**Bone and other**	87	0.45	0.20 – 1.03	0.059	0.26	0.10 – 0.69	0.007
**PgR****							
**Negative**	111	1.00			1.00		
**Positive**	204	2.07	1.04 – 4.14	0.038	1.31	0.58 – 2.97	0.515
**Her2 status****							
**Negative**	201	1.00					
**Positive**	114	0.70	0.37 – 1.32	0.272			
**Tumor differentiation****							
**Good**	46	1.00					
**Moderate**	150	0.52	0.23 – 1.17	0.116			
**Poor**	118	0.65	0.28 – 1.49	0.308			

*Age was assessed at start of tamoxifen therapy.
**Missing data not reported
Tumor differentiation was assessed through Bloom-Richardson scoring system.
Acronym: PgR: progesterone receptor

Supplementary Table S8: Univariate and multivariate analysis for the association of CALD1 staining with clinical benefit

		Univariate			Multivariate		
	**n of patients**	**OR**	**95% CI**	***P***	**OR**	**95% CI**	***P***
**CALD1**							
**Negative**	238	1.00			1.00		
**Positive**	21	0.20	0.08 – 0.57	0.002	0.21	0.08 – 0.57	0.002
**Age***							
**≤ 55 years**	109	1.00			1.00		
**> 55 years**	150	1.80	1.08 – 3.00	0.024	1.88	1.10 – 3.21	0.019
**Disease-free survival**							
**≤ 12 months**	45	1.00			1.00		
**> 12 months**	214	1.12	0.58 – 2.15	0.742	1.19	0.59 – 2.40	0.625
**Dominant site of relapse**							
**Loco-regional**	29	1.00					
**Bone**	103	0.60	0.24 – 1.48	0.269			
**Visceral**	58	0.50	0.19 – 1.32	0.163			
**Bone and other**	69	0.67	0.26 – 1.73	0.410			
**PgR**							
**Negative**	65	1.00					
**Positive**	194	1.34	0.76 – 2.37	0.315			
**Her2 status**							
**Negative**	158	1.00					
**Positive**	79	0.73	0.43 – 1.23	0.237			
**Tumor differentiation****							
**Good**	37	1.00					
**Moderate**	186	0.66	0.29 – 1.47	0.308			
**Poor**	35	0.46	0.20 – 1.06	0.069			

*Age was assessed at start of tamoxifen therapy.
**Missing data not reported
Tumor differentiation was assessed through Bloom-Richardson scoring system.
Acronym: PgR: progesterone receptor

Supplementary Table S9: Univariate and multivariate analysis for the association of CALD1 staining with objective response

		Univariate			Multivariate		
	**n of patients**	**OR**	**95% CI**	***P***	**OR**	**95% CI**	***P***
**CALD1**							
**Negative**	238	1.00			1.00		
**Positive**	21	0.49	0.11 – 2.19	0.531	0.33	0.07 – 1.61	0.171
**Age***							
**≤ 55 years**	109	1.00					
**> 55 years**	150	1.70	0.85 – 3.38	0.133			
**Disease-free survival**							
**≤ 12 months**	45	1.00			1.00		
**> 12 months**	214	1.40	0.55 – 3.55	0.474	1.55	0.58 – 4.11	0.381
**Dominant site of relapse**							
**Loco-regional**	29	1.00			1.00		
**Bone**	103	0.19	0.07 – 0.48	0.001	0.16	0.06 – 0.42	< 0.001
**Visceral**	58	0.26	0.09 – 0.72	0.010	0.24	0.08 – 0.068	0.007
**Bone and other**	69	0.27	0.10 – 0.72	0.009	0.22	0.08 – 0.61	0.004
**PgR**							
**Negative**	65	1.00					
**Positive**	194	1.37	0.62 – 3.03	0.437			
**Her2 status**							
**Negative**	158	1.00					
**Positive**	79	0.18	0.41 – 1.63	0.560			
**Tumor differentiation****							
**Good**	37	1.00					
**Moderate**	186	0.61	0.24 – 1.59	0.317			
**Poor**	35	0.90	0.35 – 2.34	0.837			

*Age was assessed at start of tamoxifen therapy.
**Missing data not reported
Tumor differentiation was assessed through Bloom-Richardson scoring system.
Acronym: PgR: progesterone receptor

Supplementary Table S12: List of proteins included in the acute phase response signaling canonical pathway

Gene name	Protein Name	Log Ratio	Expected	Location
A2M	alpha-2-macroglobulin	0.173	Up	Extracellular Space
APCS	amyloid P component, serum	0.047	Up	Extracellular Space
APOA1	apolipoprotein A-I	0.017	n/a	Extracellular Space
C3	complement component 3	0.065	Up	Extracellular Space
FGA	fibrinogen alpha chain	0.210	Up	Extracellular Space
FGB	fibrinogen beta chain	0.197	Up	Extracellular Space
FGG	fibrinogen gamma chain	0.173	Up	Extracellular Space
HP	haptoglobin	0.087	Up	Extracellular Space
HPX	hemopexin	0.059	Up	Extracellular Space
ITIH2	inter-alpha-trypsin inhibitor heavy chain 2	0.078	n/a	Extracellular Space
SERPINA1	serpin peptidase inhibitor, clade A	0.009	Up	Extracellular Space
SERPINF1	serpin peptidase inhibitor, clade F	0.040	n/a	Extracellular Space
TF	transferrin	0.080	n/a	Extracellular Space

Supplementary Table S13: Molecules found to be involved in estrogen receptor downregulation

Genes in dataset	Prediction (based on expression direction)	Log Ratio	Findings
MSN	Inhibited	0.046	Downregulates
ICAM1	Inhibited	0.169	Downregulates
COL6A2	Inhibited	0.113	Downregulates
COL6A1	Inhibited	0.107	Downregulates
COL4A2	Inhibited	0.211	Downregulates
COL4A1	Inhibited	0.170	Downregulates
CAV1	Inhibited	0.027	Downregulates
CALD1	Inhibited	0.314	Downregulates
ANXA1	Inhibited	0.279	Downregulates
C3	Activated	0.065	Upregulates
ANXA9	Activated	0.095	Upregulates
